# Factors associated with progression and sex-specific patterns in chronic subdural hematoma: a single-centre retrospective study

**DOI:** 10.3389/fneur.2025.1674327

**Published:** 2025-12-04

**Authors:** Weijian Yang, Zhuoying Du, Qifang Chen, Qiang Yuan, Pengfei Fu, Jiang Fang, Jin Hu, Gang Wu

**Affiliations:** 1 Department of Neurosurgery, Huashan Hospital, Shanghai Medical College, Fudan University, Shanghai, China; 2 National Center for Neurological Disorders, Shanghai, China; 3 Shanghai Key Laboratory of Brain Function Restoration and Neural Regeneration, Shanghai, China; 4 Neurosurgical Institute of Fudan University, Shanghai, China; 5 Shanghai Clinical Medical Center of Neurosurgery, Shanghai, China; 6 Department of Nursing, Huashan Hospital, Shanghai Medical College, Fudan University, Shanghai, China

**Keywords:** chronic subdural hematoma, sex, coagulation, inflammation, progression

## Abstract

**Objective:**

This study aims to identify determinants of chronic subdural hematoma (CSDH) progression and to evaluate potential sex-related differences.

**Methods:**

Patients with unilateral CSDH were retrospectively enrolled between January 2018 and December 2024. Data on demographics, clinical characteristics, hematoma density, hematological parameters and coagulation function were collected. Multivariable logistic regression was used to identify independent predictors. Patients were randomly divided into training and validation cohorts in a 7:3 ratio to identify factors associated with hematoma progression. Subsequently, logistic regression models were applied to both cohorts to confirm the factors influencing progression.

**Results:**

This retrospective study enrolled 1,142 patients, who were categorized into progression (*n* = 773, 67.69%) and recovery (*n* = 369, 32.31%) groups. Multivariate regression analysis revealed that international normalized ratio (INR) (OR 7.39, 95% CI 2.79–19.55), hypertension (OR 5.35, 95% CI 2.80–10.25), diabetes (OR 4.68, 95% CI 1.74–12.60), neutrophil to lymphocyte ratio (NLR) (OR 2.14, 95% CI 1.46–3.13), white blood cell count (WBC) (OR 1.16, 95% CI 1.02–1.31), and maximum hematoma density (MaHD) (OR 1.02, 95% CI 1.01–1.03) were independent risk factors for hematoma progression, whereas female gender (OR 0.61, 95% CI 0.40–0.95) was identified as a protective factor against CSDH progression. Subgroup analysis stratified by sex revealed statistically significant differences between female and male patients in hemoglobin concentration, serum albumin level, and platelet count. Nevertheless, all values remained within the respective sex-specific reference intervals.

**Conclusion:**

Elevated INR, hypertension, diabetes, NLR, WBC, and MaHD were independently associated with CSDH progression. Female sex conferred a protective effect. These findings require prospective validation.

## 1 Introduction

Chronic subdural hematoma (CSDH), defined as an encapsulated accumulation of blood products located within the subdural space, is a common neurosurgical condition among older adults (1). Population-based studies report an annual incidence of 1.7–20.6 per 100,000 person-years (2). Population ageing is projected to increase the burden of CSDH substantially on both neurosurgical services and wider healthcare systems (3). Clinical manifestations span from incidental neuroimaging findings to overt impairment of consciousness. Absence of high-level evidence underpins marked inter-hospital and international heterogeneity in management strategies (4). Asymptomatic or minimally symptomatic patients may be managed conservatively with serial clinical review and interval imaging as clinically indicated (5).

The current body of literature identifies a limited number of factors that influence the progression of hematomas. Studies have demonstrated that hematoma volume and the presence of hypodense hematoma types are significant contributors to the efficacy of conservative treatment for CSDH (5, 6). Additionally, emerging evidence indicates that the pathogenesis and progression of CSDH are closely associated with inflammatory cascades (7–9). Furthermore, coagulation profiles play a crucial role in influencing the dynamics of the disease (10, 11). Recent investigations also suggest the presence of gender disparities within CSDH populations, with female patients exhibiting distinct clinical manifestations, laboratory profiles, and therapeutic responses when compared to their male counterparts (12, 13). Nevertheless, research focusing on sex-specific differences remains limited, and the underlying mechanisms that drive these variations are not yet fully understood.

In the absence of these parameters, clinicians face considerable challenges in formulating a “wait-and-watch” strategy. This study sought to evaluate the efficacy of initial conservative treatment at the levels of the patient, coagulation, inflammatory markers, and hematoma. Furthermore, it aimed to identify factors associated with the progression of CSDH in a large, consecutive cohort of patients with unilateral CSDH. Additionally, a gender-based analysis of the disease was conducted.

## 2 Materials and methods

### 2.1 Participants and study settings

This research was conducted in accordance with the ethical standards laid down in the 1964 Declaration of Helsinki and its later amendments. Ethical approval was waived by the Ethics Committee of the Evaluation of Biomedical Research Projects of Huashan Hospital in view of the retrospective nature of the study and all the procedures being performed were part of the routine care. All patient information was de-identified, and patient consent to participate or publish was waived by the ethics committee. The study conducted a comprehensive evaluation of all inpatients and outpatients diagnosed with CSDH at Huashan Hospital, an academic teaching hospital affiliated with Fudan University, over the period from January 2018 to December 2024. The inclusion criteria encompassed: (1) patients diagnosed with primary unilateral CSDH via computed tomography (CT); and (2) individuals aged 18 years or older. The exclusion criteria included: (1) patients with a medical history of tumors, intracerebral arteriovenous malformations, arachnoid cysts, ventriculoperitoneal shunts, immunodeficiency, coagulation disorders, or chronic inflammation; (2) patients receiving immunosuppressive or anti-inflammatory treatment upon admission; and (3) patients who were lost to follow-up or had incomplete data. The study cohort was divided into two groups: progression and recovery. Recovery was defined as the resolution of CSDH on CT imaging without the need for surgical intervention. Hematoma progression was characterized by a notable decline in neurological function, including exacerbating headaches, progressive limb paralysis, or altered levels of consciousness, as well as the enlargement of the hematoma and/or midline shift observed on follow-up CT scans. The follow-up period for patients extended over six months.

### 2.2 Data collection

The study systematically gathered extensive data on patients, encompassing both demographic and clinical information. Demographic data included gender and age, while disease-related variables comprised etiology, clinical symptoms, medical history, and treatment records. Hematological parameters were assessed, including albumin levels, hemoglobin (Hb), white blood cell count (WBC), absolute neutrophil count (NEUT #), absolute lymphocyte count (LYMPH #), platelet count (PLT), neutrophil-to-lymphocyte ratio (NLR), and platelet-lymphocyte ratio (PLR). Indicators of coagulation function were also evaluated, such as prothrombin time (PT), international normalized ratio (INR), activated partial thromboplastin time (APTT), thrombin time (TT), fibrinogen (FIB), D-dimer (DDI), and fibrin degradation products (FDP). Additionally, CT findings were recorded.

**Figure 1:**
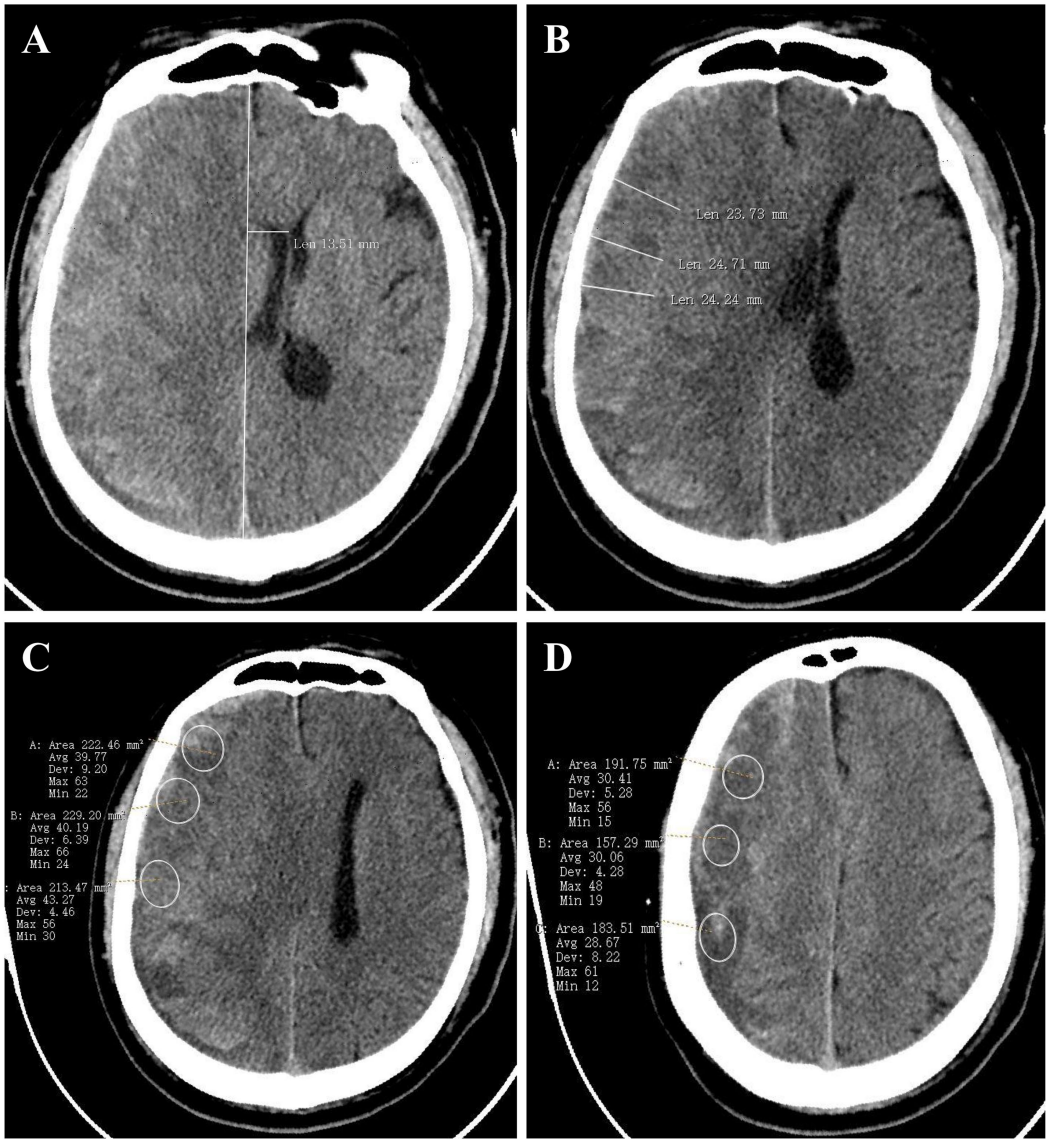
The data measurement method on CT. **(A–D)** show the measurement method of MS **(A)**, HT **(B)**, and hematoma density **(C,D)**, separately. MS, midline shift; HT, maximal hematoma thickness.

The initial CT data were collected at the time of diagnosis, including measurements of initial midline shift (MS), initial hematoma thickness (HT), minimum hematoma density (MiHD), and maximum hematoma density (MaHD). MS and HT were quantified in millimeters (mm), while CT density values were expressed in Hounsfield units (Hu). MS was defined as the line extending from the septum pellucidum, perpendicular to the midline, between the most anterior and posterior aspects of the falx cerebri. HT was measured from the brain surface, perpendicular to the tangent line of the inner compact bone. The hematoma density difference (HDD) was calculated as the difference between MaHD and MiHD. To ensure accuracy, three university-affiliated neurosurgeons verified all radiological findings, and the clinical data were anonymized using the picture archiving and communication system (PACS). The methodology for CT data measurement is illustrated in Figure 1.

### 2.3 Statistical analysis

In the univariate analysis, the distributions of variables were compared between the surgical and conservative groups, as well as between female and male participants. Categorical variables were analyzed using the chi-squared (χ^2^) test, while continuous variables were assessed using t-tests or Mann–Whitney U tests. Variables that were significant at *p* < 0.1 in the univariate analysis were included in a multivariable logistic regression model, employing a backward stepwise method based on the Akaike Information Criterion (AIC). Odds ratios (ORs) and 95% confidence intervals (CIs) were calculated. Patients were randomly divided into training and validation cohorts in a 7:3 ratio to identify factors associated with hematoma progression. Subsequently, logistic regression models were applied to both cohorts to confirm the factors influencing progression. Internal validation was conducted using the Hosmer-Lemeshow test for goodness-of-fit, calculation of the area under the receiver operating characteristic curve (AUC), assessment of model calibration, and decision curve analysis. Multicollinearity was assessed and deemed absent when the variance inflation factor (VIF) was below 5. Data processing and analysis were conducted using R version 4.3.3, in conjunction with Zstats 1.0^1^ (Accessed on July 27, 2025).

## 3 Results

### 3.1 Demographic and clinical characteristics of the study cohort

The study encompassed a total of 1,142 patients, among whom 924 (80.91%) were male (Table 1). The mean age of the participants was 68.19 years, with a standard deviation of 12.62 years. A significant proportion, 760 patients (66.55%), were within the age range of 61 to 80 years. The most frequently reported symptom was headache, occurring in 460 cases (40.28%), followed closely by dyskinesia, which was reported in 449 cases (39.32%). Additionally, 782 patients (68.48%) had a history of head trauma, and 118 patients (10.33%) reported the use of antithrombotic therapy. Hypertension was reported by 172 individuals (15.06%), while 79 individuals (6.92%) had a history of diabetes. Conservative treatment was effective for 369 patients (32.31%), whereas 773 patients (67.69%) who experienced disease progression underwent surgical intervention.

**Table 1:** Demographic and clinical characteristics of patients.

Variable	*N* (%) (total = 1,142)
Gender (female/male)	218 (19.09%)/924 (80.91%)
Age (years, mean ± SD)	68.19 ± 12.62
≤ 60	235 (20.58%)
61 < Age ≤ 80	760 (66.55%)
>81	147 (12.87%)
Neurological deficits	
Headache	460 (40.28%)
Dyskinesia	449 (39.32%)
Impaired awareness	175 (15.32%)
Aphasia	48 (4.2%)
Seizure	10 (0.88%)
History of head trauma	
Yes	782 (68.48%)
No	360 (31.52%)
Antithrombotic drugs	
Yes	118 (10.33%)
No	1,024 (89.67%)
Hypertension	
Yes	172 (15.06%)
No	970 (84.94%)
Diabetes	
Yes	79 (6.92%)
No	1,063 (93.08%)
Group	
Progression group	773 (67.69%)
Recovery group	369 (32.31%)

### 3.2 Characteristics of patients in progression and recovery group

A total of 369 patients (32.31%) were successfully managed with conservative treatment and were classified into the recovery group, whereas 773 patients (67.69%) demonstrated disease progression and were categorized into the progression group. Patients in the recovery group exhibited significantly lower rates of anti-thrombotic drug use, hypertension, and diabetes compared to those in the progression group (*p* < 0.05).

Significant differences were observed in levels of albumin, Hb, NEUT (#), Lymph (#), NLR, and PLR between the progression and recovery groups (*p* < 0.05). Notably, only the progression group exhibited NEUT (#) and NLR levels above the normal reference ranges. Regarding coagulation function, significant differences were identified in PT, INR, and FIB levels between the progression and recovery groups (*p* < 0.05), with only the INR value in the progression group exceeding the normal reference range. Additionally, statistically significant differences were found in MiHD, MaHD, and HDD between the progression and recovery groups, with higher values of MiHD and MaHD observed in the progression group (*p* < 0.05). Further details can be found in Table 2.

**Table 2:** Characteristics of patients in progression and recovery group.

Variable	Progression group	Recovery group	*P*
	*N* = 773 (67.69%)	*N* = 369 (32.31%)	
Age (years, mean ± SD)	68.70 ± 11.83	67.12 ± 14.07	0.06
≤ 60	145 (18.76%)	90 (24.39%)	
61 < Age ≤ 80	533 (68.95%)	227 (61.52%)	
>81	95 (12.29%)	52 (14.09%)	
Gender			0.08
Male	637 (82.41%)	287 (77.78%)	
Female	136 (17.59%)	82 (22.22%)	
History of head trauma			
Yes	517 (66.88%)	265 (71.82%)	0.11
No	256 (33.12%)	104 (28.18%)	
Anti-thrombotic drug			
Yes	91 (11.77%)	27 (7.32%)	0.03
No	682 (88.23%)	362 (92.68%)	
Hypertension			< 0.001
Yes	154 (19.92%)	18 (4.88%)	
No	619 (80.08%)	351 (95.12%)	
Diabetes			0.03
Yes	72 (9.31%)	7 (1.90%)	
No	701 (90.69%)	362 (98.10%)	
Neurological deficits			0.64
Headache	302 (39.07%)	158 (42.82%)	
Dyskinesia	306 (39.59%)	143 (38.75%)	
Impaired awareness	125 (16.17%)	50 (13.55%)	
Aphasia	34 (4.40%)	14 (3.79%)	
Seizure	6 (0.78%)	4 (1.08%)	
Hematological parameters			
Albumin (g/L, mean ± SD)	42.23 ± 4.01	45.79 ± 2.94	< 0.001
Hb (g/L, mean ± SD)	142.02 ± 12.33	138.95 ± 8.49	< 0.001
WBC (x10^9/L, mean ± SD)	9.88 ± 2.85	7.59 ± 1.63	0.72
NEUT (#, mean ± SD)	7.56 ± 2.50	5.08 ± 1.32	< 0.001
LYMPH (#, mean ± SD)	1.29 ± 0.43	1.74 ± 0.44	< 0.001
PLT (x10^9/L, mean ± SD)	213.58 ± 56.69	209.32 ± 25.56	0.08
NLR	6.45 ± 2.86	3.13 ± 1.22	< 0.001
PLR	184.73 ± 85.40	128.80 ± 39.40	< 0.001
Coagulation function			
PT (s, mean ± SD)	12.31 ± 2.44	12.03 ± 0.87	0.005
INR	1.25 ± 0.32	1.07 ± 0.52	< 0.001
APTT (s, mean ± SD)	25.31 ± 3.58	25.68 ± 4.44	0.17
TT (s, mean ± SD)	17.79 ± 1.57	17.81 ± 1.66	0.87
FIB (g/L, mean ± SD)	2.95 ± 0.65	3.09 ± 0.76	0.002
DDI (FEUmg/L, mean ± SD)	1.07 ± 1.15	1.04 ± 0.78	0.62
FDP (μg/ml, mean ± SD)	3.89 ± 2.26	3.97 ± 1.61	0.53
CT characteristics			
MS (mm, mean ± SD)	5.56 ± 2.57	5.57 ± 3.16	0.74
HT (mm, mean ± SD)	6.03 ± 2.37	5.93 ± 2.28	0.48
MiHD (Hu, mean ± SD)	29.86 ± 15.47	24.43 ± 13.83	< 0.001
MaHD (Hu, mean ± SD)	57.12 ± 13.76	54.77 ± 18.46	0.03
HDD (Hu, mean ± SD)	27.26 ± 14.98	30.33 ± 19.50	0.008

Hb: Hemoglobin, WBC: White Blood Cell Count, NEUT (#): Absolute Neutrophil Count, LYMPH (#): Absolute Lymphocyte Count, PLT: Platelets, NLR: Neutrophil To Lymphocyte Ratio, PLR: Platelet-lymph ratio, PT: Prothrombin Time, INR: International Normalized Ratio, APTT: Activated Partial Thromboplastin Time, TT: Thrombin Time, FIB: Fibrinogen, DDI: D-Dimer, FDP: Fibrin Degradation Products, MS: Midline Shift, HT: Hematoma Thickness, MiHD: Min Hematoma Density, MaHD: Max Hematoma Density, HDD: Hematoma Density Difference.

### 3.3 Multivariable regression analysis of CSDH progression

In a multivariable regression analysis, the coagulation parameter INR emerged as the most significant factor associated with hematoma progression (OR 7.39, 95% CI 2.79–19.55). Additionally, both hypertension (OR 5.35, 95% CI 2.80–10.25) and diabetes (OR 4.68, 95% CI 1.74–12.60) were found to be associated with hematoma progression. Inflammatory markers, such as the NLR (OR 2.14, 95% CI 1.46–3.13) and WBC (OR 1.16, 95% CI 1.02–1.31), also demonstrated significant associations with hematoma progression. Furthermore, the CT value of the hematoma, as measured by MaHD, was significantly correlated with hematoma progression (OR 1.02, 95% CI 1.01–1.03). Notably, female patients were more likely to achieve successful outcomes with conservative treatment (OR 0.61, 95% CI 0.40–0.95). Additional details can be found in Table 3.

**Table 3:** Multivariable logistic regression analysis of CSDH progression.

Variables	β (SE)	OR (95% CI)	*P*
Sex (female)	−0.49 (0.22)	0.61 (0.40–0.95)	0.03
INR	2.00 (0.50)	7.39 (2.79–19.55)	<0.001
Hypertension	1.68 (0.33)	5.35 (2.80–10.25)	<0.001
Diabetes	1.54 (0.51)	4.68 (1.74–12.60)	0.002
NLR	0.76 (0.19)	2.14 (1.46–3.13)	<0.001
WBC	0.15 (0.06)	1.16 (1.02–1.31)	0.02
MaHD	0.02 (0.01)	1.02 (1.01–1.03)	0.008

β: Regression Coefficient, SE: Standard Error, OR: Odds Ratio, CI: Confidence Interval, INR: International Normalized Ratio, NLR: Neutrophil To Lymphocyte Ratio, WBC: White Blood Cell Count, MaHD: Maximum Hematoma Density.

### 3.4 Internal validation, calibration, and decision curve analysis

**Figure 2:**
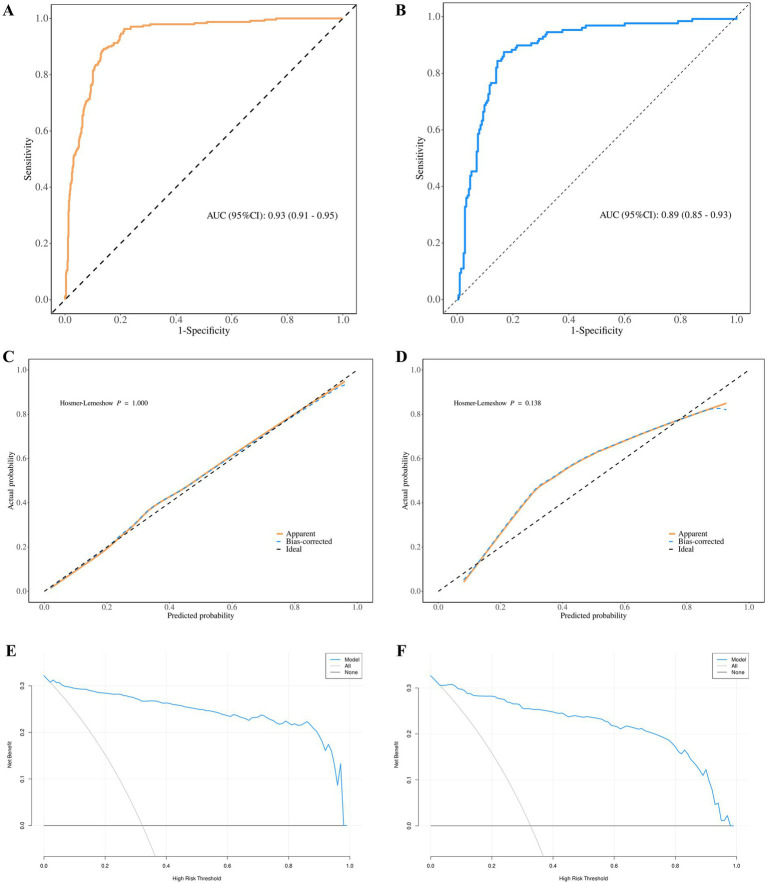
Logistic regression models for training and validation cohorts **(A,B)** ROC curves for training and validation cohorts, respectively. **(C,D)** Calibration plots for training and validation cohorts, respectively. **(E,F)** Decision curves analysis for training and validation cohorts, respectively. ROC, receiver operating characteristic; AUC, area under the curve.

To further investigate factors associated with hematoma progression, patients were randomly divided into training and validation cohorts in a 7:3 ratio. The receiver operating characteristic (ROC) curves for the factors associated with hematoma progression revealed AUC values of 0.93 (95% CI: 0.91–0.95) in the training cohort and 0.89 (95% CI: 0.85–0.93) in the validation cohort, as illustrated in Figures 2A,B. Furthermore, the calibration curves indicated a strong concordance between the observed outcomes and the predicted probabilities (Figures 2C,D). Additionally, the decision curve analysis (DCA) results demonstrated substantial clinical utility of the factors (Figures 2E,F).

### 3.5 Gender disparity analysis

Overall, the Hb concentration was significantly elevated in males compared to females, as presented in Table 4. A more detailed stratified analysis indicated that within the progression group, males exhibited a significantly higher Hb level than females (Table 5). In the recovery group, males demonstrated a significantly higher albumin concentration compared to females. Conversely, in the same group, females had a significantly higher platelet count than males (Table 6). It is noteworthy that, despite the statistically significant differences observed across various comparative analyses, all measured values remained within the established normal reference range.

**Table 4:** Characteristics of male and female patients.

Variable	Male	Female	*P*
	*N* = 924 (80.91%)	*N* = 218 (19.09%)	
Age (years, mean ± SD)	68.11 ± 12.45	68.53 ± 13.32	0.66
≤ 60	188 (20.35%)	47 (21.56%)	
61 < Age ≤ 80	625 (67.64%)	135 (61.93%)	
>81	111 (12.01%)	36 (16.51%)	
Progression			0.08
Yes	637 (68.94%)	136 (62.39%)	
No	287 (31.06%)	82 (37.61%)	
History of head trauma			0.49
Yes	628 (67.97%)	154 (70.64%)	
No	296 (32.03%)	154 (70.64%)	
Anti-thrombotic drug			
Yes	99 (10.71%)	19 (8.72%)	0.45
No	825 (89.29%)	199 (91.28%)	
Hypertension			0.89
Yes	138 (14.94%)	34 (15.60%)	
No	786 (85.06%)	184 (84.40%)	
Diabetes			0.19
Yes	59 (6.39%)	20 (9.17%)	
No	865 (93.61%)	198 (90.83%)	
Neurological deficits			0.12
Headache	382 (41.34%)	78 (35.78%)	
Dyskinesia	356 (38.53%)	93 (42.66%)	
Impaired awareness	145 (15.69%)	30 (13.76%)	
Aphasia	33 (3.57%)	15 (6.88%)	
Seizure	8 (0.87%)	2 (0.92%)	
Hematological parameters			
Albumin (g/L, mean ± SD)	43.37 ± 4.11	43.40 ± 3.83	0.92
Hb (g/L, mean ± SD)	141.78 ± 10.78	137.85 ± 12.93	**< 0.001**
WBC (x10^9/L, mean ± SD)	9.14 ± 2.71	9.12 ± 2.87	0.90
NEUT (#, mean ± SD)	6.78 ± 2.48	6.71 ± 2.47	0.74
LYMPH (#, mean ± SD)	1.44 ± 0.49	1.43 ± 0.48	0.90
PLT (x10^9/L, mean ± SD)	211.00 ± 47.99	217.29 ± 52.30	0.09
NLR	5.38 ± 2.86	5.35 ± 3.09	0.89
PLR	165.66 ± 77.14	170.90 ± 82.76	0.37
Coagulation function			
PT (s, mean ± SD)	12.22 ± 1.94	12.22 ± 2.55	0.99
INR	1.19 ± 0.28	1.07 ± 0.52	0.32
APTT (s, mean ± SD)	25.40 ± 3.82	25.57 ± 4.12	0.55
TT (s, mean ± SD)	17.78 ± 1.61	17.86 ± 1.53	0.50
FIB (g/L, mean ± SD)	2.99 ± 0.69	3.00 ± 0.71	0.79
DDI (FEUmg/L, mean ± SD)	1.08 ± 1.10	0.99 ± 0.75	0.27
FDP (μg/ml, mean ± SD)	3.91 ± 2.17	3.91 ± 1.57	0.96
CT characteristics			
MS (mm, mean ± SD)	5.57 ± 2.73	5.60 ± 2.92	0.87
HT (mm, mean ± SD)	6.00 ± 2.34	5.97 ± 2.33	0.86
MiHD (Hu, mean ± SD)	28.40 ± 15.08	26.88 ± 15.51	0.18
MaHD (Hu, mean ± SD)	56.46 ± 15.32	55.94 ± 16.12	0.66
HDD (Hu, mean ± SD)	28.06 ± 16.56	29.06 ± 16.94	0.43

Hb: Hemoglobin, WBC: White Blood Cell Count, NEUT (#): Absolute Neutrophil Count, LYMPH (#): Absolute Lymphocyte Count, PLT: Platelets, NLR: Neutrophil To Lymphocyte Ratio, PLR: Platelet-lymph ratio, PT: Prothrombin Time, INR: International Normalized Ratio, APTT: Activated Partial Thromboplastin Time, TT: Thrombin Time, FIB: Fibrinogen, DDI: D-Dimer, FDP: Fibrin Degradation Products, MS: Midline Shift, HT: Hematoma Thickness, MiHD: Min Hematoma Density, MaHD: Max Hematoma Density, HDD: Hematoma Density Difference.

The bold values are used solely for visual emphasis to highlight data points that are statistically significant.

**Table 5:** Characteristics of male and female patients in progression group.

Variable	Male	Female	*P*
	*N* = 637 (82.41%)	N = 136 (17.59%)	
Age (years, mean ± SD)	68.60 ± 11.87	69.13 ± 11.72	0.64
≤ 60	188 (20.35%)	47 (21.56%)	
61 < Age ≤ 80	625 (67.64%)	135 (61.93%)	
>81	111 (12.01%)	36 (16.51%)	
History of head trauma			0.76
Yes	424 (66.56%)	93 (68.38%)	
No	213 (33.44%)	43 (31.62%)	
Anti-thrombotic drug			
Yes	76 (11.93%)	15 (11.03%)	0.88
No	561 (88.07%)	121 (88.97%)	
Hypertension			0.99
Yes	127 (19.94%)	27 (19.85%)	
No	510 (80.06%)	109 (80.15%)	
Diabetes			0.36
Yes	56 (8.79%)	16 (11.76%)	
No	581 (91.21%)	120 (88.24%)	
Neurological deficits			0.15
Headache	255 (40.03%)	47 (34.56%)	
Dyskinesia	249 (39.09%)	57 (41.91%)	
Impaired awareness	104 (16.33%)	21 (15.44%)	
Aphasia	23 (3.61%)	11 (8.09%)	
Seizure	6 (0.94%)	0 (0.00%)	
Hematological parameters			
Albumin (g/L, mean ± SD)	42.17 ± 4.02	42.46 ± 3.94	0.45
Hb (g/L, mean ± SD)	142.80 ± 12.18	138.36 ± 12.41	**< 0.001**
WBC (x10^9/L, mean ± SD)	9.82 ± 2.82	10.15 ± 2.95	0.22
NEUT (#, mean ± SD)	7.54 ± 2.50	7.70 ± 2.50	0.48
LYMPH (#, mean ± SD)	1.30 ± 0.44	1.28 ± 0.41	0.57
PLT (x10^9/L, mean ± SD)	212.42 ± 55.81	219.00 ± 60.57	0.22
NLR	6.41 ± 2.79	6.64 ± 3.14	0.39
PLR	183.42 ± 84.18	190.86 ± 90.96	0.36
Coagulation function			
PT (s, mean ± SD)	12.30 ± 2.26	12.37 ± 3.17	0.74
INR	1.25 ± 0.31	1.25 ± 0.40	0.80
APTT (s, mean ± SD)	25.31 ± 3.54	25.32 ± 3.79	0.98
TT (s, mean ± SD)	17.76 ± 1.58	17.92 ± 1.52	0.30
FIB (g/L, mean ± SD)	2.95 ± 0.66	2.93 ± 0.61	0.80
DDI (FEUmg/L, mean ± SD)	1.08 ± 1.23	1.03 ± 0.62	0.61
FDP (μg/ml, mean ± SD)	3.90 ± 2.41	3.81 ± 1.37	0.67
CT characteristics			
MS (mm, mean ± SD)	5.54 ± 2.59	5.60 ± 2.52	0.81
HT (mm, mean ± SD)	6.01 ± 2.35	6.13 ± 2.44	0.60
MiHD (Hu, mean ± SD)	30.27 ± 15.32	27.94 ± 16.06	0.11
MaHD (Hu, mean ± SD)	57.44 ± 13.73	55.63 ± 13.86	0.17
HDD (Hu, mean ± SD)	27.17 ± 14.87	27.69 ± 15.51	0.72

Hb: Hemoglobin, WBC: White Blood Cell Count, NEUT (#): Absolute Neutrophil Count, LYMPH (#): Absolute Lymphocyte Count, PLT: Platelets, NLR: Neutrophil To Lymphocyte Ratio, PLR: Platelet-lymph ratio, PT: Prothrombin Time, INR: International Normalized Ratio, APTT: Activated Partial Thromboplastin Time, TT: Thrombin Time, FIB: Fibrinogen, DDI: D-Dimer, FDP: Fibrin Degradation Products, MS: Midline Shift, HT: Hematoma Thickness, MiHD: Min Hematoma Density, MaHD: Max Hematoma Density, HDD: Hematoma Density Difference.

The bold values are used solely for visual emphasis to highlight data points that are statistically significant.

**Table 6:** Characteristics of male and female patients in recovery group.

Variable	Male	Female	*P*
	*N* = 287 (78%)	*N* = 82 (22%)	
Age (years, mean ± SD)	67.00 ± 13.62	67.54 ± 15.64	0.76
≤ 60	70 (24.39%)	20 (24.39%)	
61 < Age ≤ 80	180 (62.72%)	47 (57.32%)	
>81	37 (12.89%)	15 (18.29%)	
History of head trauma			0.65
Yes	204 (71.08%)	61 (74.39%)	
No	83 (28.92%)	21 (25.61%)	
Anti-thrombotic drug			
Yes	23 (8.01%)	4 (4.88%)	0.47
No	264 (91.99%)	78 (95.12%)	
Hypertension			0.15
Yes	11 (3.83%)	7 (8.54%)	
No	276 (96.17%)	75 (91.46%)	
Diabetes			0.05
Yes	3 (1.05%)	4 (4.88%)	
No	284 (98.95%)	78 (95.12%)	
Neurological deficits			0.34
Headache	127 (44.25%)	31 (37.80%)	
Dyskinesia	107 (37.28%)	36 (43.90%)	
Impaired awareness	41 (14.29%)	9 (10.98%)	
Aphasia	10 (3.48%)	4 (4.88%)	
Seizure	2 (0.70%)	2 (2.44%)	
Hematological parameters			
Albumin (g/L, mean ± SD)	46.03 ± 2.85	44.96 ± 3.10	**0.004**
Hb (g/L, mean ± SD)	139.50 ± 6.14	137.01 ± 13.78	0.12
WBC (x10^9/L, mean ± SD)	7.64 ± 1.62	7.41 ± 1.67	0.25
NEUT (#, mean ± SD)	5.09 ± 1.33	5.07 ± 1.27	0.93
LYMPH (#, mean ± SD)	1.75 ± 0.43	1.70 ± 0.46	0.34
PLT (x10^9/L, mean ± SD)	207.85 ± 22.15	214.46 ± 34.66	**0.04**
NLR	3.10 ± 1.20	3.21 ± 1.29	0.47
PLR	126.24 ± 34.36	137.78 ± 52.73	0.06
Coagulation function			
PT (s, mean ± SD)	12.05 ± 0.90	11.96 ± 0.74	0.36
INR	1.05 ± 0.12	1.15 ± 1.07	0.11
APTT (s, mean ± SD)	25.59 ± 4.40	25.99 ± 4.60	0.47
TT (s, mean ± SD)	17.82 ± 1.70	17.77 ± 1.54	0.81
FIB (g/L, mean ± SD)	3.08 ± 0.74	3.12 ± 0.84	0.67
DDI (FEUmg/L, mean ± SD)	1.07 ± 0.73	0.94 ± 0.92	0.17
FDP (μg/ml, mean ± SD)	3.94 ± 1.53	4.07 ± 1.86	0.54
CT characteristics			
MS (mm, mean ± SD)	5.62 ± 3.01	5.60 ± 3.50	0.96
HT (mm, mean ± SD)	5.99 ± 2.32	5.72 ± 2.14	0.35
MiHD (Hu, mean ± SD)	24.24 ± 13.65	25.11 ± 14.48	0.62
MaHD (Hu, mean ± SD)	54.29 ± 18.19	56.45 ± 19.39	0.35
HDD (Hu, mean ± SD)	30.05 ± 19.68	31.34 ± 18.95	0.60

Hb: Hemoglobin, WBC: White Blood Cell Count, NEUT (#): Absolute Neutrophil Count, LYMPH (#): Absolute Lymphocyte Count, PLT: Platelets, NLR: Neutrophil To Lymphocyte Ratio, PLR: Platelet-lymph ratio, PT: Prothrombin Time, INR: International Normalized Ratio, APTT: Activated Partial Thromboplastin Time, TT: Thrombin Time, FIB: Fibrinogen, DDI: D-Dimer, FDP: Fibrin Degradation Products, MS: Midline Shift, HT: Hematoma Thickness, MiHD: Min Hematoma Density, MaHD: Max Hematoma Density, HDD: Hematoma Density Difference.

The bold values are used solely for visual emphasis to highlight data points that are statistically significant.

## 4 Discussion

This retrospective study aimed to investigate the factors associated with the progression of CSDH. The cohort comprised 1,142 patients, of whom 19.09% were female. Multivariable regression analysis identified that INR, hypertension, diabetes, NLR, WBC, and MaHD were independently associated with CSDH progression. Notably, female sex emerged as a protective factor against hematoma progression. Further subgroup analysis revealed statistically significant differences between female and male patients in Hb levels, albumin concentrations, and platelet counts, although all values remained within the normal reference range.

CSDH predominantly affects the elderly population, with our findings indicating that individuals aged 61 to 80 years constitute 66.55% of the cases. As the global population continues to age, the prevalence of CSDH is anticipated to increase, potentially imposing substantial burdens on healthcare systems and families (14). Treatment options for CSDH include conservative management for asymptomatic patients with small hematomas and surgical intervention for more severe cases. The risk of treatment failure with both strategies ranges from 5 to 50%, often resulting in persistence or worsening of the condition (15). Therefore, identifying factors associated with CSDH progression could provide valuable insights for neurologists in developing effective follow-up and management strategies.

The increasing use of antithrombotic medications appears to contribute to the development of CSDH (16). Meta-analyses indicate that approximately 17.33% of CSDH patients are receiving antithrombotic therapy at the time of presentation (17). In our study, 10.33% of patients were undergoing antithrombotic therapy. A case–control study demonstrated that the use of antithrombotic drugs is associated with a higher risk of subdural hematoma (10). It is well-established that the INR reflects the coagulation function in patients. Research indicates that when INR values in patients with CSDH exceed 1.25, the condition may deteriorate or even result in mortality (18). Our study indicates that the mean INR in the progression group is 1.25, which exceeds the normal range, whereas in the recovery group, it is 1.07, falling within the normal range. Multivariate analysis identifies INR as a significant factor associated with hematoma progression.

The trauma-induced inflammatory response facilitates hematoma expansion by increasing vascular permeability, promoting capillary formation, enhancing fibrinolytic activity, and recruiting immune cells (19). Both hypertension and diabetes potentially exert pro-inflammatory effects. Chronic hypertension induces inflammatory alterations in arterial vessels, ultimately resulting in endothelial damage and vascular stiffness (20). Diabetes mellitus is characterized by elevated tissue levels of inflammatory markers (21). A national study has identified hypertension and diabetes as risk factors for the occurrence and progression of CSDH (8). Our multivariate analysis further corroborates that hypertension and diabetes are risk factors for CSDH progression, with odds ratios of 5.35 and 4.68, respectively. The NLR serves as an indicator of the dynamic interplay between the innate immune response, represented by neutrophils, and the adaptive cellular immune response, represented by lymphocytes, during illness and various pathological conditions. Research indicates that patients with CSDH exhibit significantly higher NLR values compared to healthy controls (22). Further studies have established a correlation between elevated postoperative NLR and an increased risk of recurrence (23). In our cohort, patients experiencing progression had mean NLR values exceeding the normal range, which were significantly higher than those observed in patients undergoing recovery. Multivariate regression analysis identified NLR as a risk factor for hematoma progression, with an odds ratio of 2.14.

CSDH is marked by blood products older than two to three weeks (24). As these blood products degrade over time, the hematoma undergoes liquefaction and becomes encapsulated by a fibrous membrane. In the absence of recent hemorrhage, CSDH typically manifests as a homogeneously hypodense lesion (25). According to Zhang et al., hypodense hematomas are independent predictors of a “wait and watch” management strategy (26). Foppen et al. identified hematoma volume and hypodense hematoma type were associated with successful conservative treatment outcomes (5). The underlying theory posits that hypodense hematomas lack active bleeding components (5). The high density observed within SDH may be attributed to the acute hemorrhage of existing CSDH (27). Our research indicates that MaHD is a contributing factor to hematoma progression.

Our multivariate analysis identified female sex as a protective factor against CSDH progression, a finding that warrants further discussion regarding potential underlying mechanisms. The observed sex disparity may be attributed to a combination of biological and socio-behavioral factors. From a biological perspective, the influence of sex hormones, particularly estrogen, is a leading hypothesis. Estrogen has been demonstrated to possess neuroprotective, anti-inflammatory, and pro-angiogenic properties (28). It may attenuate the inflammatory cascade and promote more stable vascular repair within the neo membranes of CSDH, thereby reducing the propensity for hematoma expansion and progression. In contrast, testosterone in males has been linked to a more pronounced pro-inflammatory state, which could exacerbate the local pathology (29). Furthermore, well-established physiological differences, such as generally lower levels of blood pressure and a potentially different vascular reactivity profile in pre-menopausal women, might contribute to a less favorable environment for hematoma progression (30). From a socio-behavioral standpoint, epidemiological studies consistently report that male sex is a risk factor for traumatic brain injury, the primary etiology of CSDH (12). This is often linked to higher rates of risk-taking behaviors, occupational hazards, and a greater incidence of falls in elderly males. Consequently, the initial insult and the subsequent pathophysiological response might differ between sexes.

While our subgroup analysis revealed significant differences in hemoglobin, albumin, and platelet counts between sexes, all values remained within normal limits, suggesting that these common clinical parameters may not fully explain the protective effect. The mechanisms are likely multifactorial and rooted in deeper hormonal and molecular pathways. Future prospective studies incorporating dedicated biomarker analyses are essential to validate these hypotheses and elucidate the precise causes of the sex-specific disparities observed in CSDH outcomes.

This study has several important limitations. Its retrospective, single-center design inherently limits causal inference and may introduce selection bias and unmeasured confounding. Moreover, as the data were derived from a single institution, the generalizability of our findings to other populations or healthcare settings remains uncertain. Another limitation is the lack of subgroup analyses comparing different surgical techniques or conservative management strategies. Future studies should adopt multicenter, prospective designs that incorporate broader outcome measures and stratify patients based on specific clinical, radiological, and treatment variables—such as the number of burr holes or timing of intervention. Such rigorously designed research would be essential for validating the factors associated with CSDH progression and sex-specific disparities, ultimately helping to refine personalized treatment approaches.

## 5 Conclusion

This study examined the risk factors contributing to the progression of CSDH and emphasized the significance of sex-specific variations. The factors identified as being associated with CSDH progression included INR, hypertension, diabetes, NLR, WBC, and MaHD. Additionally, female sex was found to be a protective factor against hematoma progression.

## Data Availability

The raw data supporting the conclusions of this article will be made available by the authors, without undue reservation.

## Footnotes

1
www.zstats.net
